# Supplementary material for: A randomized, controlled study to assess if allopathic-osteopathic collaboration influences stereotypes, interprofessional readiness, and doctor-patient communication
Source: PLoS One. 2022 Dec 1;17(12):e0278171. doi: 10.1371/journal.pone.0278171 (PMC9714813; doi:10.1371/journal.pone.0278171)
Supplement: S1 Appendix — (DOCX) [file pone.0278171.s002.docx]

**Statistical Analysis Plan**

**Assessing Stereotypes and Interprofessional Readiness**

For the two primary outcomes, since there are two students’ response per pair, multivariable linear mixed effect models with pair-level random effect will be used to assess the difference in SSRQ and RIPLS scores among difference combinations (MD/DO, MD/MD, DO/DO). The analysis will also be adjusted for certain covariates, e.g. students’ grade, whether response was from MD or DO, etc.

For the secondary outcome, Pearson’s correlation or Spearman’s correlation test will be conducted to assess the association between SSRQ and RIPL. In the subset analysis, two sample t-test or non-parametric Wilcoxon rank-sum test will be used to assess the difference between MD and DO patients within the MD/DO pair.

**Patient experience**

Since each pair of medical students are expected to see 2-4 patients together, there will be multiple patient responses for each pair of medical students. For the primary outcome, multivariable linear mixed effect model will be used to assess the difference in patient experience score among combinations. Pair-level random effect will be included in the model to account for within-pair correlation and variation. Possible adjustment will also be considered, like patients’ demographics and clinical characteristics.

For the secondary aim, multivariable linear mixed effect model will be used to assess the association between SSRQ and RIPLS scores from the students and patient experience score. The outcome will be the patient experience scores (multiple scores per pair), while the key explanatory variable will be average SSRQ or RIPLS score from the two students in the pair. Similar to the analysis above, within-pair correlation will be controlled by adding a pair-level intercept, while confounding factors like patients’ characteristics will be adjusted in the model.

**Power and Sample Size Considerations**

The power will be determined based on the primary aim only. Bonferroni correction was used to adjust for multiple comparisons. Since there were 3 primary outcomes (2 in student survey, 1 in patient survey), the significance level was adjusted to be 0.05/3=0.0167.

For the primary aims, one-way ANOVA test was used to calculate the power. Assuming an effect size of 0.33 (variation among the 3 combinations), the design of n=40 pairs in each combination, totaling n=120 pairs of medical students, achieves 80% power to detect the differences among the means versus the alternative of equal means using an F test with a 0.0167 significance level. The repeated measure design described above will achieve even higher power by collecting multiple data points per pair.

**Randomization Schedule and adaptive-design concern**

Randomization schedule will be provided by Yanjun Chen, Principal biostatistician in BERD, ICTS before the trial get started. The allocation for each combination (MD/DO, MD/MD, DO/DO) will be 1:1:1. Data will be analyzed at interim during the trial to examine the effect. Possible conditional power will be calculated during the interim analysis to see if we need to increase the sample size, or terminate the trial early.
