# Supplementary material for: A randomized, controlled study to assess if allopathic-osteopathic collaboration influences stereotypes, interprofessional readiness, and doctor-patient communication
Source: PLoS One. 2022 Dec 1;17(12):e0278171. doi: 10.1371/journal.pone.0278171 (PMC9714813; doi:10.1371/journal.pone.0278171)
Supplement: Attachment — Submitted filename: Requested change.docx [file pone.0278171.s004.docx]

Requested change #1: change article type from clinical trial to clinical study

- The authors wholeheartedly agree with the requested change. To this end, we have removed any remaining language in the manuscript referring to the study as a trial. The word “trial” was replaced by “study” on lines 216, 253 and 264.
- We have left the consort diagram in the manuscript (requested previously by your editorial staff), as we believe this was a great suggestion that will help readers understand the study design.
- We have removed the Consort checklist, as this is no longer needed.
