# Supplementary material for: A randomized, controlled study to assess if allopathic-osteopathic collaboration influences stereotypes, interprofessional readiness, and doctor-patient communication
Source: PLoS One. 2022 Dec 1;17(12):e0278171. doi: 10.1371/journal.pone.0278171 (PMC9714813; doi:10.1371/journal.pone.0278171)
Supplement: Attachment — Submitted filename: Response to Reviewers.docx [file pone.0278171.s005.docx]

**Line-item response are provided in red below.**

1.  During your revisions, please confirm whether the wording in the title is correct and update it in the manuscript file and online submission information if needed. Specifically, alter the title from "A Randomized, Controlled Study to Assess how Allopathic-Osteopathic Collaboration Effects Stereotypes, Interprofessional Readiness, and Doctor-Patient Communication" to "A Randomized, Controlled Study to Assess how Allopathic-Osteopathic Collaboration Affects Stereotypes, Interprofessional Readiness, and Doctor-Patient Communication"

Per the last correspondence and reviewer request, the title has been changed to “A Randomized, Controlled Study to Assess if Allopathic-Osteopathic Collaboration Influences Stereotypes, Interprofessional Readiness, and Doctor-Patient Communication”. To the best of my knowledge, all fields in the submission portal were updated

2. Can you please upload an additional copy of your revised manuscript that does not contain any tracked changes or highlighting as your main article file.  This will be used in the production process if your manuscript is accepted.  Please amend the file type for the file showing your changes to Revised Manuscript w/tracked changes. Please follow this link for more information: [http://blogs.PLOS.org/everyone/2011/05/10/how-to-submit-your-revised-manuscript/](http://blogs.plos.org/everyone/2011/05/10/how-to-submit-your-revised-manuscript/)

Complete

3. Please include a caption for figure 1.

Figure 1 caption added

4. Please ensure that you refer to Table 2 and 4 in your text as, if accepted, production will need this reference to link the reader to the Table.

Thank you for identifying this error. In text reference to table 2 and table 4 have been added

**June 18^th^ 2022 responses can be found below**

**We were able to comply with all requests for the returned manuscript. Line-item response are provided in red below. We would once again like to thank the editorial staff for the high-quality feedback.**

1. Please ensure that your manuscript meets PLOS ONE's style requirements, including those for file naming. The PLOS ONE style templates can be found at

Style changes made

2. During your revisions, please confirm whether the wording in the title is correct and update it in the manuscript file and online submission information if needed. Specifically, alter the title from "A Randomized, Controlled Study to Assess how Allopathic-Osteopathic Collaboration Effects Stereotypes, Interprofessional Readiness, and Doctor-Patient Communication" to "A Randomized, Controlled Study to Assess how Allopathic-Osteopathic Collaboration Affects Stereotypes, Interprofessional Readiness, and Doctor-Patient Communication""""

Title changed for clarity

3. You indicated that ethical approval was not necessary for your study. We understand that the framework for ethical oversight requirements for studies of this type may differ depending on the setting and we would appreciate some further clarification regarding your research. Could you please provide further details on why your study is exempt from the need for approval and confirmation from your institutional review board or research ethics committee (e.g., in the form of a letter or email correspondence) that ethics review was not necessary for this study? Please include a copy of the correspondence as an ""Other"" file."""

A file titled “IRB” containing IRB self-exemption documentation with signed/dated IRB correspondence confirming said exemption has been uploaded.

4. Thank you for stating the following in the Competing Interests/Financial Disclosure section:

“The University of California Medical Humanities Consortium Summer Research Stipend recipient was awarded to Ian Jones and Michael LoBasso for their work on this project.
Alpesh Amin reported serving as PI or co-I of clinical trials sponsored by NIH/NIAID, NeuroRx Pharma, Pulmotect, Blade Therpeutics, Novartis, Takeda, Humanigen, Eli Lilly, PTC Therapeutics, OctaPharma, Fulcrum Therapeutics, Alexion. He has served as speaker and/or consultant for BMS, Pfizer, BI, Portola, Sunovion, Mylan, Alexion, AstraZeneca, Novartis, Nabriva, Paratek, Bayer, Tetraphase, Achogen LaJolla, PeraHealth, HeartRite, Aseptiscope, Sprightly.”

We note that one or more of the authors are employed by a commercial company:

a. Please provide an amended Funding Statement declaring this commercial affiliation, as well as a statement regarding the Role of Funders in your study. If the funding organization did not play a role in the study design, data collection and analysis, decision to publish, or preparation of the manuscript and only provided financial support in the form of authors' salaries and/or research materials, please review your statements relating to the author contributions, and ensure you have specifically and accurately indicated the role(s) that these authors had in your study. You can update author roles in the Author Contributions section of the online submission form.

Please also include the following statement within your amended Funding Statement.

“The funder provided support in the form of salaries for authors [insert relevant initials], but did not have any additional role in the study design, data collection and analysis, decision to publish, or preparation of the manuscript. The specific roles of these authors are articulated in the ‘author contributions’ section.”

If your commercial affiliation did play a role in your study, please state and explain this role within your updated Funding Statement.

b. Please also provide an updated Competing Interests Statement declaring this commercial affiliation along with any other relevant declarations relating to employment, consultancy, patents, products in development, or marketed products, etc.

Within your Competing Interests Statement, please confirm that this commercial affiliation does not alter your adherence to all PLOS ONE policies on sharing data and materials by including the following statement: "This does not alter our adherence to  PLOS ONE policies on sharing data and materials.” (as detailed online in our guide for authors <http://journals.plos.org/plosone/s/competing-interests>) . If this adherence statement is not accurate and  there are restrictions on sharing of data and/or materials, please state these. Please note that we cannot proceed with consideration of your article until this information has been declared.

Please include both an updated Funding Statement and Competing Interests Statement in your cover letter. We will change the online submission form on your behalf.

Our cover letter is a signed pdf document, as such, the updated Funding Statement and Competing Interests Statement are provided below. Please note that, none of the authors are employed by a commercial company

**Funding:** Funding for biostatistician consulting services was provided by the UCI Hospital Medicine Program and UC Department of Medicine. The UC Irvine Center for Statistical Consulting is partially supported by grant UL1 TR001414 from the National Center for Advancing Translational Sciences, National Institutes of Health (NIH), through the Biostatistics, Epidemiology and Research Design Unit. The content is solely the responsibility of the authors and does not necessarily represent the official views of the NIH. Additionally, the University of California Medical Humanities Consortium Summer Research Stipend recipient was awarded to Ian Jones and Michael LoBasso for their work on this project. The funder provided support in the form of a summer research stipend for authors I.J. and M.L. but did not have any additional role in the study design, data collection and analysis, decision to publish, or preparation of the manuscript. One or more authors have declared potential conflicts of interest, as described below. However, these affiliations do not alter authors adherence to PLOS ONE policies on sharing data and materials, and no commercial affiliation played a role in any study-related activities.

**Competing Interests:** Alpesh Amin has served as PI or co-I of clinical trials sponsored by NIH/NIAID, NeuroRx Pharma, Pulmotect, Blade Therpeutics, Novartis, Takeda, Humanigen, Eli Lilly, PTC Therapeutics, OctaPharma, Fulcrum Therapeutics, Alexion. He has served as speaker and/or consultant for BMS, Pfizer, BI, Portola, Sunovion, Mylan, Alexion, AstraZeneca, Novartis, Nabriva, Paratek, Bayer, Tetraphase, Achogen LaJolla, PeraHealth, HeartRite, Aseptiscope, Sprightly, Ferring, Spero, Seres, Eli Lilly. All authors declare no conflicts or competing interests for this work. The specific roles of authors are articulated in the ‘author contributions’ section.

5. We note that you have stated that you will provide repository information for your data at acceptance. Should your manuscript be accepted for publication, we will hold it until you provide the relevant accession numbers or DOIs necessary to access your data. If you wish to make changes to your Data Availability statement, please describe these changes in your cover letter and we will update your Data Availability statement to reflect the information you provide

The following information has been uploaded as compressed files: “complete dataset” and “qualitative analysis”. The former file includes all survey response, and the later file includes the thematic analysis that was performed. We request that these data files be included with the manuscript as supplemental materials. We have amended the text in the additional information section of the submission portal to read, “All relevant data are within the manuscript and its Supporting Information files”

6. Please include captions for your Supporting Information files at the end of your manuscript, and update any in-text citations to match accordingly. Please see our Supporting Information guidelines for more information: <http://journals.plos.org/plosone/s/supporting-information>.

Captions for Supporting Information files, as references in the manuscript, have been added.

Reviewers' comments:

Reviewer's Responses to Questions

**Comments to the Author**

Please use the space provided to explain your answers to the questions above. You may also include additional comments for the author, including concerns about dual publication, research ethics, or publication ethics. (Please upload your review as an attachment if it exceeds 20,000 characters)

Reviewer #1: The authors present a study about differing perceptions of DO and MD physicians. I have some concerns about the methodology and lack of detail in the paper.

There is a need for some upfront explanation of the DO/MD distinction – the term osteopathic physician is not universally understood (I think it is a US-centric system that does not exist in other countries). In other countries, osteopath would not normally be regarded as a ‘physician’. These titles and the associated roles need to be better described in the introduction.

This is an excellent point.

There is an important distinction between osteopathic physicians trained in the United States and osteopaths trained in other countries. Osteopathic physicians trained in the United States are licensed medical doctors that have full practice rights. Moreover, these rights are often honored when osteopathic physicians leave to practice in a different county. In contrast, most foreign-trained osteopaths (which also identify as DOs) are issued a Diploma of Osteopathy, not a Doctor of Osteopathic Medicine. They are often not licensed to prescribe medications or perform surgeries and are instead trained primarily in osteopathic manipulative techniques.

An abbreviated version of the above context has been added to the manuscript. Also note that potential self-selection bias is noted in the discussion as a limitation.

Some further detail on the methods would be useful, for example:

• The participants are described as students ‘volunteering’ in the clinic – in what circumstances do students volunteer? Are these formal clinical placements? Was the task (patient interview and examination) part of routine training or an additional task specific to this study?

This is a fair critique, and additional clarification has been added to the methods.

Medical student volunteers (both allopathic and osteopathic) are those who have elected to work a non-mandatory, unpaid shift. They are tasked with responsibilities analogous to what would be expected from a medical student during their standard clinical training. These responsibilities include, chart checking patients prior to the encounter, independently performing a history and physical exam, and then presenting their assessment and plan to an attending physician. These activities are part of the standard operating procedure for the clinic and are not unique to the study itself.

An abbreviated version of the above context has been added to the manuscript

• Who was responsible for recruiting students and patients? How were patient participants identified and recruited?

As described under “Ethics and dissemination of information”, participation was limited to adults aged 18 or older, but otherwise included all medical students volunteering at An Lanh Clinic and patients visiting An Lanh Clinic on days where staffing was sufficient to support the predetermined randomization schedule.

Manuscript wording has been modified to make the above more explicit.

• Were all the students from the same medical school/s? What is the curriculum structure in terms of patient communication training?

This study compares osteopathic and allopathic students. As such we are comparing students from two different medical schools. To make this clearer, the methods have been updated to explicitly state that all medical students from two different medical schools (an allopathic school and an osteopathic school) were eligible to participate.

It should also be noted that the discussion section states that “only two institutions participated in the study” as one of the major limitations.

The qualitative data items are described very briefly only – are these questions incorporated into the surveys? This needs further explanation.

Questions were incorporated into the surveys. This has been made explicit in the revised draft.

To what extent is the pairing for the activity likely to have impacted on perceptions? Is it possible that the individuals within each pair had differing views even before the task? This is more likely I think, given that there seems to have been no briefing or preparation of the pairs prior to the task.

Since the pairing itself was the intervention, differing views before clinic were expected and controlled for by randomization. It appears that perhaps the reviewer is raising a concern related to pre- vs post-intervention views. That is, we did not measure stereotypes and interprofessional readiness at baseline, and then reassess their views after the competition of the clinic shift. While a reasonable concern, performing both pre- and post-intervention assessment would have been difficult for several reasons. First, it would have potentially biased the results, with questionnaires calling attention to the intervention, effectively unblinding students to the pairing intervention. It also would have likely resulted in survey fatigue and reduced student compliance. Lastly, given that shifts lasted 4-5 hours, collecting pre- and post-intervention surveys could have introduced potential recency bias and/or masked effects by way of students remembering their prior answers.

The design of this study seems to be somewhat flawed in that it seems to assume that simply doing a task together is influencing perceptions – I would think that as an educational opportunity, expanding the task to including briefing and debriefing components would be more useful and more likely to generate differences/educational benefit.

The fact we did not measure whether a briefing or debriefing component influences student perceptions is a feature of this study, not a flaw. The study gives us insight into the potential effect that working together can have, independent of formal training. Indeed, numerous studies have investigated attempts to implement formal educational didactic sessions relating to stereotypes and or interprofessional training. This study is unique in that it looks at how exposure alone influenced these factors.

While the authors respectfully disagree with the above critique, we believe the reviewer is right to be interested in how a briefing or debriefing component might influence student or patient perceptions. Potential directions for future studies include comparing the relative effectiveness of formal didactic education (which could include a briefing and/or debriefing component) to that of early clinical exposure. With respect to questions relating to MD/DO collaboration, it is not known whether formal education is inferior/superior/non-inferior to early clinical exposure. It is also possible that a combined approach that included both a formal didactic component and real-world exposure might have synergistic effects. Additional text has been added to the end of the manuscript describing some of these potential future directions.

In terms of the impact of the study in adding knowledge to the field, my previous comment reflects a concern I have about the overall value of this study.

See above response

Some specifics:

• Acronyms used in the abstract need to be given in full in the first instance

MD and DO have been defined

• Language in the abstract needs to be refined – first section reads awkwardly

Abstract has been simplified

• Page 5 line 67 should read ‘a’ rather than ‘an’

Addressed
