# Supplementary material for: A randomized, controlled study to assess if allopathic-osteopathic collaboration influences stereotypes, interprofessional readiness, and doctor-patient communication
Source: PLoS One. 2022 Dec 1;17(12):e0278171. doi: 10.1371/journal.pone.0278171 (PMC9714813; doi:10.1371/journal.pone.0278171)
Supplement: Attachment — Submitted filename: Response to Reviewers.docx [file pone.0278171.s006.docx]

Please see line item responses to review concerns below. Overall, we believe the suggested edits improved the quality of the manuscript. We thank both the editorial office and reviewers for their time and consideration.

**Thank you for the opportunity to review this revision of the manuscript. Overall, the manuscript reads satisfactorily and the changes have strengthened the manuscript. There needs to be greater links to the literature in the Discussion/Conclusion section. Much of the commentary here is no referenced or linked back to the literature.**

In an effort to reduce manuscript bloat, our discussion focused on contextualizing findings, rather than making commentary on the literature as a whole. There is also a paucity of data on MD/DO interaction in the literature, as evidenced by the delays in finding qualified reviewers for this manuscript. Taken together, these factors lead to a discussion with less citations than might otherwise be included in a prospective study of similar quality. It should also be noted that, the original manuscript write up had contained close to twice as many citations. However, after critical appraisal, many of these references were removed, as they contained questionable methods and/or provided low quality data. Nevertheless, we understand the reviewers' concern and have attempted to add additional citations to our discussion. In total 6 new references have been added to the discussion section of the manuscript.

**Page 5, line 85 - it would be good to separate into paragraphs the data collected from the students and that collected from the patients.**

The requested change has been made

**Page 6 - line 94- clarify that the Student Stereotypes Rating Questionnaire measures stereotypes of one's own profession and of other health professions. This isn't entirely clear at present.**

The requested change has been made

**Page 6 - line 103, provide an example of a dual degree**

The requested change has been made

**Page 7, line 131 - who was the control group for the study? This isn't clear from the description provided.**

Control was not discussed in this section. This information has been added. In brief, the allocation to work in an MD/DO group was the intervention, and allocation to work in the MD/MD or DO/DO group was the control. Generally speaking, MD and DO students don’t have a chance to work together during the first two years of their medical education. Thus, allocation to an MD/DO group during an early medical education represents a deviation from standard practice.

**Page 9 - line 152 - please provide additional data about the thematic analysis, particularly who performed the analysis, their experience with the analytical approach etc. The COREQ (**[**https://www.equator-network.org/reporting-guidelines/coreq/**](https://www.equator-network.org/reporting-guidelines/coreq/)**) will be helpful in guiding the additional detail required. On reviewing the Results, it would appear that these are more consistent with a content analysis approach given the commentary on frequency etc. (e.g. "compared to the DO/DO group, the MD/MD group was more than twice as likely to report concerns"**

The reviewer may be correct that in the end our analysis ended up being more content than thematic. Although, the authors believe that a case could be made for our use of the term “thematic analysis”, particularly given the fact that, in an inductive approach, we were able to generate themes based on the preliminary coded data. However, in the interest of addressing the reviewers concern, we have re-written the qualitative methods section to put what we did in a conventional and summative content analysis framework.

Regarding COREQ, which is a 32 item checklist, we believe that it would likely not be possible or constructive to employ given the limited scope of the data. The qualitative component was a small part of this study meant to add context to the quantitative data obtained through randomization and the use of validated questionnaires.

We have also tried to address concerns regarding the experience of the individuals performing the analysis. However, this concern is a bit hard to address without unblinding and/or including the CV of author JS. The manuscript now states explicitly which author led this portion of the analysis. We are also happy to elaborate further if the reviewer/editor believes that additional information about qualifications is needed.

**Page 13, line 227 - may be better to describe the MD/MD combination as demonstrating lower RIPLS scores rather than 'worst'**

The requested change has been made
